# Acute kidney injury in hospitalized patients who underwent percutaneous kidney biopsy for histological diagnosis of their renal disease

**DOI:** 10.1186/s12882-019-1514-8

**Published:** 2019-08-13

**Authors:** Henrique Pinheiro Konigsfeld, Tatiana Garcia Viana, Suzy Cristine Pereira, Thais Oliveira Claizoni Dos Santos, Gianna Mastroianni Kirsztajn, Agostinho Tavares, Marcelino de Souza Durão Junior

**Affiliations:** 1 Nephrology Division, Universidade Federal de São Paulo, São Paulo, SP Brazil; 2 Kidney Transplant Unit, Hospital Israelita Albert Einstein, São Paulo, SP Brazil

**Keywords:** Acute kidney injury, Glomerulonephritis, Hemoglobin, Kidney biopsy, HDL-c

## Abstract

**Background:**

Performing a kidney biopsy is necessary to accurately diagnose diseases such as glomerulonephritis and tubulointerstitial nephritis, among other such conditions. These conditions predispose patients to chronic kidney disease, as well as acute kidney injury (AKI). Notably, most epidemiological studies describing AKI have not investigated this patient population.

**Methods:**

Included patients admitted to the nephrology ward of a tertiary hospital who underwent percutaneous kidney biopsy. AKI was diagnosed based on the Kidney Disease: Improving Global Outcomes criteria.

**Results:**

Of the 223 patients investigated, 140 (62.8%) showed AKI. Of these, 91 (65%), 19 (13.6%), and 30 (21.4%) presented with AKI classified as stages 1, 2, and 3, respectively. The primary indication for performing biopsy was nephrotic syndrome or nephrotic proteinuria (73 [52.1%] in the AKI vs. 51 [61.4%] in the non-AKI group, *p* = 0.048). Focal segmental glomerulosclerosis was the most prevalent primary disease (24 [17.1%] in the AKI vs. 15 [18.0%] in the non-AKI group, *p* = 0.150). Multivariate analysis of risk factors associated with AKI showed hemoglobin levels (odds ratio [OR] 0.805, 95% confidence interval [CI] 0.681–0.951, *p* = 0.011), serum high-density lipoprotein cholesterol levels (HDL-c, OR 0.970, 95% CI 0.949–0.992, *p* = 0.008), and baseline serum creatinine levels (OR 2.703, 95% CI 1.471–4.968, *p* = 0.001) were significantly associated with AKI.

**Conclusions:**

We observed a high prevalence of AKI in hospitalized patients who underwent kidney biopsy to investigate their renal disease, particularly glomerulonephritis. Higher levels of hemoglobin and serum HDL-c were associated with a lower risk of AKI.

## Background

Acute kidney injury (AKI) is a risk factor for the development of chronic kidney disease (CKD) and chronic end-stage renal disease (ESRD). AKI is also associated with increased short- and long-term mortality rates [1]. AKI is a complex and heterogeneous clinical syndrome observed mainly in elderly patients with several comorbidities. These patients are usually admitted to intensive care units (ICU), and AKI is primarily attributed to sepsis in such cases [2]. Percutaneous renal biopsy is usually contraindicated in these patients to prevent complications, and they are usually diagnosed with “acute tubular necrosis”.

In contrast, some kidney diseases such as glomerulonephritis and tubulointerstitial nephritis, among other such clinical conditions require histopathological analysis of kidney tissue specimens to accurately determine diagnosis, disease activity and chronicity [3, 4]. Notably, most epidemiological studies have investigated patients with AKI have not included this patient population in the analysis. For example, a meta-analysis analyzed the worldwide incidence of AKI observed that studies primarily included patients admitted to the ICU and those who underwent cardiac surgery [5].

Therefore, we investigated the prevalence and risk factors associated with AKI in hospitalized patients who underwent kidney biopsy for histopathological diagnosis.

## Methods

### Study design and patients

This was a retrospective cohort study, included patients admitted to the Nephrology ward of Hospital São Paulo, Universidade Federal de São Paulo, São Paulo, Brazil. Patients were eligible if submitted to percutaneous kidney biopsy performed with aged ≥16 years as indicated by the nephrologists. Exclusion criteria were kidney transplant recipients. In our center, all percutaneous native kidney biopsies were performed in patients admitted exclusively to the nephrology ward. We did not perform native kidney biopsy in outpatient setting. Data were obtained, between January 2008 and December 2014, from patients’ medical records and the electronic hospital database. The follow-up period underwent 12-month after the kidney biopsy.

### Demographic and clinical data

We analyzed the following variables: age, sex, race, body mass index (BMI), major comorbidities such as hypertension, diabetes mellitus (DM), cancer, cardiovascular disease (CVD including heart failure, coronary artery disease, stroke, and peripheral artery disease), and systemic lupus erythematosus (SLE).

We evaluated the use of diuretics, antibiotics, statins, angiotensin-converting enzyme inhibitors (ACEI), and/or angiotensin receptor blockers (ARB), nonsteroidal anti-inflammatory drugs (NSAID), and contrast media (recent use as well as administration during hospitalization). Previous use of corticosteroids was also recorded.

Indications for renal biopsy were nephrotic syndrome (characterized by edema, hypoalbuminemia [< 3.5 g/L], dyslipidemia, and proteinuria ≥3.5 g/24 h [h]), or nephrotic-range proteinuria (proteinuria ≥3.5 g/24 h), rapidly progressive glomerulonephritis (RPGN, characterized by hematuria and/or proteinuria on urinalysis associated with rapid decline in renal function indicated by progressively increasing serum creatinine levels, which may occur over days, weeks, or months prior to renal biopsy), sub-nephrotic proteinuria (isolated proteinuria > 1.0 g/24 h), dysmorphic hematuria associated with some grade of proteinuria, renal dysfunction if unknown origin, and renal manifestations of systemic diseases.

### Evaluation performed upon admission

The following parameters were evaluated upon admission: hemoglobin, serum sodium, potassium, ionic calcium, phosphorus, and albumin levels, pH and bicarbonate levels, serum total cholesterol and its fractions, serum triglycerides, serum creatinine and urea levels, as well as 24-h urinary protein excretion. The glomerular filtration rate (GFR) was estimated using the Modification of Diet in Renal Disease Study Group (MDRD) equation [6].

All patients underwent kidney and urinary tract ultrasonography. The length of the kidneys was recorded in all patients.

Optical microscopy analysis and immunofluorescence studies were performed for histopathological diagnostic confirmation as well electron microscopy, when indicated. All analysis was performed by a single renal pathologist.

We evaluated the serum creatinine level upon admission, during hospitalization, and specific time-points after discharge until 12 months after the kidney biopsy. The reading that showed the lowest level during hospitalization was considered the baseline serum creatinine level because laboratory data prior to hospitalization were unavailable. The highest serum creatinine level during hospitalization was used to diagnose AKI.

### Acute kidney injury

We defined and classified AKI observed during hospitalization based on the Kidney Disease: Improving Global Outcomes (KDIGO) criteria using only serum creatinine levels [7]. AKI was defined as an increase in serum creatinine ≥0.3 mg/dL over ≤48 h or a ≥ 1.5-fold increase in serum creatinine over baseline levels within the 7 days. AKI was classified into 3 stages as follows: stage 1: ≥1.5 to 1.9-fold increase in serum creatinine levels or an increase of 0.3 mg/dL compared with baseline levels, stage 2: ≥2.0 to 2.9-fold increase in serum creatinine levels compared with baseline levels, and stage 3: > 3.0-fold increase in serum creatinine levels compared with baseline creatinine levels or serum creatinine levels > 4.0 mg/dL with an increase of at least 0.5 mg/dL, or the initiation of renal replacement therapy.

### Outcomes

The following outcomes were assessed: kidney function (evaluated by serum creatinine levels), need for dialysis initiation, and mortality within 12 months after kidney biopsy.

### Statistical analysis

Data were expressed as mean and standard deviation for normally distributed variables and as median and quartiles (25–75%) for variables showing non-normal distribution. Parametric distribution was confirmed using the Shapiro-Wilk test. We used the Student’s t-test or the Mann-Whitney test for numerical variables and the Chi-square test for comparison between nominal variables.

For covariance analyze repeated measures of creatinine and for intergroup comparison of data, we used the Generalized Estimation Equations and applied the autoregressive structure correlation matrix [1] and gamma distribution with log link.

Variables that were significantly associated with AKI on univariate analysis (*p* < 0.05) were subjected to multivariate logistic regression analysis for variables such as age and sex.

A *p* value < 0.05 was considered statistically significant. SPSS software, version 20.0 (IBM Corp, USA, 2011) was used for statistical analysis.

## Results

The study included 267 patients, of which 44 (16.5%) patients were diagnosed with ESRD during admission and were excluded from the study. Of the remaining patients (N = 223), 140 (62.8%) were diagnosed with AKI during hospitalization. No significant intergroup difference was observed in sex (men, 68 [48.6%] in the AKI vs. 41 [49.4%] in the non-AKI group, *p* = 0.508). Patients with AKI were older than patients without AKI (41.2 ± 17.7 vs. 35.3 ± 14.4 years, *p =* 0.03) with similar BMI (25.7 ± 5.4 in the AKI vs. 24.5 ± 4.0 kg/m2 in the non-AKI group, p = 0.291). Most patients were white in both groups (87 [62.8%] in the AKI vs. 51 [61.5%] in the non-AKI group, *p =* 0.482).

No statistically significant differences were observed between the AKI and non-AKI groups, respectively in the percentage of patients with hypertension (64 [45.7%] vs. 30 [36.1%], *p =* 0.096), patients with CVD (9 [6.4%] vs. 3 [3.6%], *p* = 0.279), and patients with a history of cancer (5 [3.6%] vs. 1 [1.2%], *p* = 0.273). DM was more prevalent in the AKI group (17 [12.1%] vs. 3 [3.6%], *p* = 0.023).

A lower percentage of patients in the AKI group used ACEIs/ARBs (57 [40.7%] vs. 45 [54.2%], *p* = 0.038). Additionally, a larger number of patients in the AKI group received a potentially nephrotoxic antibiotic (16 [11.4%] vs. 2 [2.4%], *p* = 0.012). No statistically significant difference was observed between the AKI and non-AKI groups with respect to the use of diuretics (42 [30.0%] vs. 32 [38.6%], *p* = 0.130), statins (31 [22.1%] vs. 23 [27.7%], *p* = 0.227), NSAIDs (19 [13.6%] vs. 9 [10.8%], *p* = 0.347), and corticosteroids (34 [3.0%] vs. 28 [33.7%], *p* = 0.091). No patient underwent contrast examination during hospitalization.

The primary indications for renal biopsy were nephrotic-range proteinuria or nephrotic syndrome (73 [52.1%] in the AKI vs. 51 [61.4%] in the non-AKI group, *p* = 0.048), renal dysfunction of undetermined etiology (23 [16.4%] in the AKI vs. 5 [6.0%] in the non-AKI group, *p* = 0.001), and hematuria with non-nephrotic-range proteinuria (11 [7.8%] in the AKI vs. 11 [13.2%] in the non-AKI group, *p* = 1,000). RPGN was an indication exclusively in the AKI group (18 [12.9%] vs. 0, *p* < 0.001).

Primary renal disease was the most common type of disease observed in both groups (86 [61.4%] in the AKI vs. 67 [80.7%] in the non-AKI group, *p* = 0.125). The primary diagnoses were focal segmental glomerulosclerosis (FSGS) (24 [17.1%] in the AKI vs. 15 [18.0%] in the non-AKI group, *p* = 0.150) and minimal change disease (MCD) (21 [15%] in the AKI vs. 15 [18%] in the non-AKI group, *p* = 0.317). Secondary diseases predominated in the AKI group (54 [38.6%] vs. 16 [19.3%], *p* < 0.001). SLE was the main cause of secondary renal disease, with classes III and IV being the most prevalent (Table 1).

**Table 1:** Intergroup comparison of baseline demographic, clinical data upon admission, indications for kidney biopsy and histopathological examination findings

	Total	AKI	Non-AKI	*p* value
N (%)	223 (100)	140 (62.8)	83 (37.2)	
Male sex (%)	109 (48.9)	68 (48.6)	41 (49.4)	0.508
Age (years)	39.8 ± 16.8	41.2 ± 17.7	35.3 ± 14.4	0.030
White race (%)	138 (61.9)	87 (62.8)	51 (61.4)	0.482
BMI (kg/m^2^)	25.3 ± 5.1	25.7 ± 5.4	24.5 ± 4.0	0.291
Comorbidities, n (%)				
Hypertension	94 (42.3)	64 (45.7)	30 (36.1)	0.096
Diabetes	20 (9.0)	17 (12.1)	3 (3.6)	0.023
CVD	12 (5.4)	9 (6.4)	3 (3.6)	0.279
Cancer	6 (2.7)	5 (3.6)	1 (1.2)	0.273
Medications administered (%)				
ACEIs/ARBs	102 (45.9)	57 (40.7)	45 (54.2)	0.038
Diuretics	74 (33.3)	42 (30.0)	32 (38.6)	0.130
Statins	54 (24.3)	31 (22.1)	23 (27.7)	0.227
NSAIDs	28 (12.6)	19 (13.6)	9 (10.8)	0.347
Antibiotics	18 (8.1)	16 (11.4)	2 (2.4)	0.012
Previous use of corticosteroids (%)	62 (27.9)	34 (24.3)	28 (33.7)	0.091
Indications for biopsy (%)				
Nephrotic-range proteinuria or NS	124 (55.6)	73 (52.1)	51 (61.4)	0.048
Renal dysfunction of undetermined etiology	28 (12.6)	23 (16.4)	5 (6.0)	0.001
Hematuria with proteinuria (non-nephrotic range)	22 (9.9)	11 (7.8)	11 (13.2)	1.000
Renal manifestations of systemic disease	22 (9.9)	13 (9.3)	9 (10.8)	0.394
RPGN	18 (8.0)	18 (12.9)	0	< 0.001
Isolated proteinuria (non-nephrotic range)	9 (4.0)	2 (1.4)	7 (8.4)	0.096
Type of kidney disease (%)				
Primary	153 (68.6)	86 (56.2)	67 (43.8)	0.125
Secondary	70 (31.4)	54 (77.1)	16 (22.9)	< 0.001
Histopathological diagnosis (%)				
Primary				
FSGS	39 (17.5)	24 (17.1)	15 (18.0)	0.150
MCD	36 (16.1)	21 (15.0)	15 (18.0)	0.317
MGN	33 (14.8)	16 (11.4)	17 (20.5)	0.862
IgA	19 (8.5)	12 (8.6)	7 (8.4)	0.251
CGN	12 (5.4)	7 (5.0)	5 (6.0)	0.564
MPGN	6 (2.7)	1 (0.7)	5 (6.0)	0.102
Others	8 (3.6)	5 (3.6)	3 (3.6)	0.480
Secondary				
SLE III/IV	30 (13.5)	19 (13.6)	11 (13.2)	0.144
ADGN	7 (3.1)	7 (5.0)	0	< 0.001
AIN	7 (3.1)	6 (4.3)	1 (1.2)	0.059
SLE V	5 (2.2)	4 (2.9)	1 (1.2)	0.180
Hypertensive Nephrosclerosis	4 (1.8)	3 (2.1)	1 (1.2)	0.317
ATN	2 (0.9)	2 (1.4)	0	< 0.001
Amyloidosis/MM	2 (0.9)	1 (0.7)	1 (1.2)	1.000
Diabetes	2 (0.9)	2 (1.4)	0	< 0.001
ANCA-associated GN	2 (0.9)	2 (1.4)	0	< 0.001
Others	9 (4.9)	8 (5.7)	1 (1.2)	0.020

*ACEIs/ARBs* angiotensin-converting enzyme inhibitors/angiotensin II receptor blockers, *AKI* acute kidney injury, *BMI* body mass index, *CVD* cardiovascular disease, *NSAIDs* non-steroidal anti-inflammatory drugs, *ADGN* acute diffuse glomerulonephritis (post-infectious), *AIN* acute interstitial nephritis, *AKI* acute kidney injury, *ANCA*–*associated GN* antineutrophil cytoplasmic antibody-associated glomerulonephritis, *ATN* acute tubular necrosis, *CGN* chronic glomerulonephritis, *DM* diabetes mellitus, *FSGS* focal segmental glomerulosclerosis, *IgA* immunoglobulin A glomerulonephritis, *MCD* minimal change disease, *MGN* membranous glomerulonephritis, *MM* multiple myeloma, *MPGN* membranoproliferative glomerulonephritis, *NS* nephrotic syndrome, *RPGN* rapidly progressive glomerulonephritis, *SLE* systemic lupus erythematosus–classes III, IV and V

Laboratory data and measurements of renal length are presented in Table 2. Statistically significant differences were observed between the AKI and non-AKI groups with respect to the following variables: serum hemoglobin (11.7 ± 2.7 vs. 13.4 ± 2.1 g/dL, p < 0.001), serum sodium (137 ± 3.7 vs. 138 ± 2.6 mEq/L, *p* = 0.047), serum urea (76.0 [55.0–115.0] vs. 57.0 [39.0–77.3] mg/dL, p < 0.001), serum bicarbonate (21.4 ± 5.5 vs. 25.7 ± 4.0 mEq/L, p < 0.001), and serum HDL-c (43.5 ± 16.2 vs. 56.3 ± 28.5 mg/dL, p < 0.001). No statistically significant intergroup difference was observed in renal length.

**Table 2:** Intergroup comparison of laboratory and ultrasonographic data

	Total (223)	AKI (140)	Non-AKI (83)	p value
Laboratory tests				
Serum hemoglobin (g/dL)	12.3 ± 2.6	11.7 ± 2.7	13.4 ± 2.1	< 0.001
Serum ionized calcium (mg/dL)	1.20 ± 0.1	1.21 ± 0.11	1.2 ± 0.07	0.454
Serum phosphorus (mg/dL)	4.35 ± 1.2	4.5 ± 1.4	4.0 ± 0.9	0.07
Serum sodium (mEq/L)	137.6 ± 3.4	137 ± 3.7	138 ± 2.6	0.047
Serum potassium (mEq/L)	4.33 ± 0.7	4.4 ± 0.8	4.2 ± 0.5	0.060
Serum urea (mg/dL)	63.0 (36.5–94.5)	76.0 (55.0–115.0)	57.0 (39.0–77.3)	< 0.001
pH	7.3 (7.3–7.3)	7.3 (7.3–7.3)	7.3 (7.3–7.4)	0.126
Serum bicarbonate (mEq/L)	22.7 ± 5.4	21.4 ± 5.5	25.7 ± 4.0	< 0.001
Total serum cholesterol (mg/dL)	256 (191–388)	247 (189–357)	415 (273–506)	0.127
Serum HDL-c (mg/dL)	47.9 ± 22.0	43.5 ± 16.2	56.3 ± 28.5	< 0.001
Serum LDL-c (mg/dL)	158 (108–223)	150 (113–220)	159 (100–239)	0.862
Serum triglycerides (mg/dL)	216.0 (136–320)	239.0 (140–325)	186.0 (127–319)	0.231
Serum albumin (mg/dL)	2.4 (1.7–3.5)	2.4 (1.6–3.5)	2.2 (1.8–3.5)	0.783
Proteinuria (g/24 h)	4.50 (2.10–7.64)	4.50 (1.89–8.09)	4.56 (2.36–6.93)	0.969
Renal size (cm)				
Right kidney	11.0 ± 1.4	11.1 ± 1.4	10.8 ± 1.6	0.397
Left kidney	11.2 ± 1.3	11.2 ± 1.4	11.1 ± 1.0	0.741

*AKI* acute kidney injury, *HDL-c* high-density lipoprotein cholesterol, *LDL-c* low-density lipoprotein cholesterol

Table 3 shows an intergroup comparison of serum creatinine levels across different time-points in the study. We observed that the AKI group showed higher serum creatinine levels than the non-AKI group *(p* < 0.001) across the entire follow-up period. Compared with peak levels during admission, we observed a decrease in serum creatinine levels in the AKI group after 12 months (*p* < 0.05).

**Table 3:** Intergroup comparison of serum creatinine levels (mg/dL) during follow-up

	AKI		Non-AKI		
	Median (25–75%)	N	Median (25–75%)	N	p value
Admission levels	1.98 (1.28–3.25)	140	0.88 (0.66–1.16)	83	< 0.001
Baseline levels	1.65 (1.13–2.34)	140	0.80 (0.61–1.08)	83	< 0.001
Peak levels	2.83 (1.86–4.41)	140	0.97 (0.75–1.24)	83	< 0.001
At discharge	1.96 (1.32–2.73)	139	0.88 (0.71–1.21)	83	< 0.001
1 month	1.72 (1.00–2.60)	119	0.92 (0.72–1.31)	66	< 0.001
3 months	1.27 (0.80–2.35)	84	0.81 (0.68–1.31)	44	< 0.001
9 months	1.28 (0.85–2.34)	83	0.90 (0.74–1.66)	48	< 0.001
12 months	1.24 (0.84–2.31)*	73	0.86 (0.68–1.47)	47	< 0.001

*AKI* acute kidney injury

* p < 0.05 vs. highest intragroup level during hospitalization

Among patients diagnosed with AKI (62.8%), 91 (65.0%) were classified as showing stage I disease, 19 (13.6%) as stage 2, and 30 (21.4%) as stage 3 disease.

Dialysis was performed in 19 (8.5%) patients in the AKI group and of these 19, 9 (4.0%) remained dependent on dialysis therapy at the time of discharge. All patients were dialysis-free at the end of 12 months. Notably, 8 patients (3.6%) died and 5 of them died of infections during hospitalization. Table 4 shows the multivariate analysis of factors associated with the development of AKI.

**Table 4:** Multivariate logistic regression analysis of factors associated with the development of acute kidney injury

	Odds ratio (CI 95%)	p value
Male sex	0.670 (0.307–1.458)	0.312
Age (years)	1.006 (0.983–1.029)	0.641
Serum hemoglobin levels	0.805 (0.681–0.951)	0.011
Serum HDL-c levels	0.970 (0.949–0.992)	0.008
Baseline serum creatinine levels	2.703 (1.471–4.968)	0.001
Constant	34.836	0.010

*AKI* acute kidney injury, *CI* confidence interval, *HDL-c* high-density lipoprotein cholesterol

## Discussion

This study showed a high prevalence of AKI in hospitalized patients who underwent kidney biopsy for renal disease primarily caused by glomerulonephritis. Most patients showed primary renal disease, and the most common indications for renal biopsy were nephrotic-range proteinuria or nephrotic syndrome. Among the primary diseases, FSGS showed the highest prevalence. Among all secondary diseases, lupus glomerulonephritis was the most common.

Epidemiological studies investigating AKI should necessarily describe patients’ baseline serum creatinine levels. In this study, we defined the lowest serum creatinine level during hospitalization as an estimate of baseline renal function, given the lack of previous values. Retrospective baseline serum creatinine levels (assuming GFR = 75 mL/min/1.73 m^2^) based on KDIGO criteria may miss the occurrence of AKI in the community [7]. Moreover, our study group comprised patients predominantly with glomerulonephritis, which tends to demonstrate an insidious onset and progression. Occasionally, in a few cases, AKI can be diagnosed in clinical practice only by analyzing temporal changes in serum creatinine levels. Our findings concur with this observation in that progressive improvement in renal function occurred in the AKI group throughout the study period.

We could not rule out the existence of a pre-renal component of AKI, particularly in patients with less severe AKI (stage 1). Despite the long-term protective effect of ACEI/ARB in renal diseases in patients with proteinuria, their use may cause acute and reversible renal dysfunction, particularly when used concomitantly with diuretics and in patients with other conditions causing hypovolemia [8, 9]. Interestingly, we observed a higher percentage of patients using ACEI/ARB in the non-AKI group. In contrast to data reported in the literature, the effect of AKI on glomerular hemodynamics (decreased filtration pressure) and the potential negative effect on renal function was not evident in the present study [8, 10]. For example, a recent study investigating patients with hypertension using ACEI/ARB and diuretics showed AKI was associated with CKD and poor cardiac performance [11]. Another study involving new users of ACEI/ARB reported a low incidence of AKI and showed AKI was more likely to be associated with the individual clinical characteristics than with the use of the medication itself [12].

Proteinuria and low serum albumin levels are risk factors for AKI [13, 14]. This is particularly evident in patients diagnosed with MCD, notably elderly patients with hypertension (showing arteriolar nephrosclerosis) and patients with more severe degrees of nephrotic syndrome [15]. In our cohort, no significant differences were observed in 24-h proteinuria, serum albumin levels, and the number of patients diagnosed with major proteinuria-causing diseases (FSGS, MCD and Membranous Glomerulonephritis) between the AKI and non-AKI groups. Previous studies have shown that DM is also an independent risk factor for AKI, mainly secondary to microvascular dysfunction [16, 17]. Although the AKI group included a greater number of patients with DM, it was not an independent risk factor in our study. Our study included younger patients than those studied previously, and owing to the multiple disease diagnosed, AKI could be attributed to other mechanisms as well.

Pre-existing kidney dysfunction serves as an independent risk factor for AKI in conditions such as sepsis and contrast-induced nephropathy, as well as in patients undergoing cardiac surgery or solid organ transplantation, and in patients admitted to the ICU [18–22]. This factor was also relevant in our study. Patients with CKD are at a higher risk of AKI secondary to the role of several etiopathogenetic contributors such as activation of transforming growth factor beta, action of hypoxia-inducible factors, mitochondrial and endothelial dysfunction, oxidative stress, chronic inflammation, and alterations in renal blood flow autoregulation observed in this patient population [23–25].

Anemia and AKI are occasionally associated. A few studies have shown anemia serves as an independent risk factor for AKI [26–28]. Previous studies have shown anemia was a risk factor for contrast-induced nephropathy in patients undergoing coronary angiography [29], postoperatively in patients undergoing hip arthroplasty [30], in patients admitted to the ICU [31], and postoperatively in patients undergoing cardiac surgery [32]. In these cases, AKI associated with anemia could be attributed to a reduction in the oxygen supply to renal tissues and the consequent aggravation of pre-existing ischemia occurring in hospitalized patients [32–34].

In our study, we observed a higher level of serum hemoglobin was associated with a lower incidence of AKI. Several studies have shown an association between anemia and AKI; however, it is unclear whether higher levels of serum hemoglobin could reduce the incidence of AKI. It could be deduced that early intervention, for example, through the use of recombinant human erythropoietin, could reduce the risk of AKI in specific diseases, such as in glomerulonephritis. Previous studies have shown the role of erythropoietin in patients admitted to the ICU and in those undergoing cardiac surgery was controversial and in a few cases the benefit was independent of hematocrit and serum hemoglobin levels [35].

Altered lipid metabolism is common in patients with renal disease, particularly in those with glomerulonephritis and nephrotic syndrome. Hypercholesterolemia and hypertriglyceridemia are the most common findings in such cases; however, low serum HDL-c levels may occur. Resolution of nephrotic syndrome usually tends to reverse these changes [36].

HDL-c possesses antioxidant properties, thereby reducing endothelial damage and the risk of atherosclerosis [37]. The MDRD study showed a low serum HDL-c level was an independent risk factor associated with a faster decline in GFR. In experimental AKI models, HDL-c showed anti-inflammatory actions and reduced ischemia and reperfusion injury [38, 39]. Roveran Genga et al. showed low levels of serum HDL-c in patients with sepsis were associated with a higher incidence of sepsis-associated AKI [40]. Another study reported by Smith et al. demonstrated higher levels of preoperative serum HDL-c decreased the incidence of AKI after cardiac surgery [41]. Arora et al. observed low levels of serum HDL-c were associated with AKI after revascularization surgery for chronic limb ischemia [42].

In our cohort, we observed an association between higher levels of serum HDL-c and lower incidence of AKI. We inferred perhaps intensive drug therapy for dyslipidemia in addition to exercise and dietary strategies could improve patients’ lipid profile and consequently reduce the incidence of AKI, particularly in patients with glomerulonephritis.

The following are the limitations of our study: [1] The lack of outpatient baseline serum creatinine values is the main limitation, since some cases of non-recovering AKI could be missed. [2] Because it is a retrospective and single-center study, a bias cannot be ruled out. [3] Histopathological heterogeneity and dropouts during follow-up are other limitations. [4] We do not have information regarding the treatment for each specific disease diagnosed by biopsy. Despite these limitations, the high prevalence of AKI observed in these subjects is concerning because this patient population is often excluded from epidemiological studies investigating AKI.

## Conclusions

We observed a high prevalence of AKI in hospitalized patients who underwent kidney biopsy to investigate their renal disease, particularly glomerulonephritis. Higher levels of hemoglobin and serum HDL-c were associated with lower risks of AKI.

## Data Availability

The data that support the findings of this study are available from Universidade Federal de São Paulo (UNIFESP), but restrictions apply to the availability of these data, which were used under license for the current study, and so are not publicly available. Data are however available from the authors upon reasonable request and with permission of Universidade Federal de São Paulo (UNIFESP).

## Abbreviations

ACEI: angiotensin-converting enzyme inhibitors
AKI: Acute kidney injury
ARB: angiotensin receptor blockers
BMI: body mass index
CKD: chronic kidney disease
CVD: cardiovascular disease
DM: diabetes mellitus
ESRD: chronic end-stage renal disease
FSGS: focal segmental glomerulosclerosis
GFR: glomerular filtration rate
HDL-c: high-density lipoprotein cholesterol
ICU: intensive care units
KDIGO: Kidney Disease: Improving Global Outcomes
MCD: minimal change disease
MDRD: Modification of Diet in Renal Disease Study Group
NSAID: nonsteroidal anti-inflammatory drugs
RPGN: rapidly progressive glomerulonephritis
SLE: systemic lupus erythematosus
